# A Rare Case of Monostotic Fibrous Dysplasia of the Femoral Neck With Pathological Fracture: A Case Report

**DOI:** 10.7759/cureus.49085

**Published:** 2023-11-20

**Authors:** Adarsh Jayasoorya, Gajanan Pisulkar, Nitin Samal, Shounak Taywade, Shrut N Vasavada

**Affiliations:** 1 Department of Orthopedics, Jawaharlal Nehru Medical College, Datta Meghe Institute of Higher Education and Research, Wardha, IND; 2 Department of Orthopaedic Surgery, Jawaharlal Nehru Medical College, Datta Meghe Institute of Higher Education and Research, Wardha, IND

**Keywords:** total hip arthroplasty (tha), histopathology, radiography, bone lesion, monostotic, fibrous dysplasia

## Abstract

Fibrous dysplasia is a rare benign bone disorder characterized by the replacement of normal bone with fibroblastic and osteoblastic tissue. We present a case of monostotic fibrous dysplasia in a 25-year-old male patient. The case highlights the clinical presentation, radiographic features, and management approach for this condition. This report aims to contribute to the understanding of fibrous dysplasia and its management options. A 25-year-old male presented with a chief complaint of persistent left hip pain. The pain was described as a dull ache, associated with difficulty in weight-bearing activities. There was no history of trauma or constitutional symptoms. Physical examination revealed externally rotated left lower limb. Range of motion of the left hip could not be assessed due to pain, with no neurological deficits noted. Initial imaging included plain radiographs of the right femur, which demonstrated a radiolucent lesion with a ground-glass appearance and cortical thinning. Magnetic resonance imaging of both hip joints reveals an irregular T2 hyperintense and T1 hypointense lesion involving the left femoral neck; moreover, few tiny cystic spaces are seen within the lesion. Part of the lesion is extending into the superior-lateral aspect of the femoral head and surrounding bone marrow edema with minimal left hip joint effusion, features suggestive of a primary bony tumour. Plain computed tomography (CT) of the hip joint and pelvis was suggestive of an expansile lytic lesion with thin bony septation within and thick sclerotic margin in the left femoral head and greater trochanter associated with sub-capital femoral neck fracture suggestive of bone neoplasm (? giant cell tumour > simple bone cyst). A bone biopsy was performed, and histopathological examination confirmed the diagnosis of fibrous dysplasia, with characteristic woven bone and fibrous stroma. In this case, after confirming the diagnosis, the patient was managed with total hip arthroplasty on the left side. Monostotic fibrous dysplasia is a rare benign bone disorder that can present with various clinical manifestations. Timely diagnosis through a combination of clinical, radiographic, and histopathological assessments is crucial. Management should be tailored to the patient's symptoms.

## Introduction

Fibrous dysplasia (FD) is a rare benign bone disorder characterized by the replacement of normal bone with fibroblastic and osteoblastic tissue. First described by Lichtenstein in 1938, this condition presents a unique challenge in both diagnosis and management due to its diverse clinical manifestations and unpredictable disease course [1]. FD can occur as either a monostotic form, affecting a single bone, or a polyostotic form, involving multiple bones. Monostotic FD is more common, accounting for approximately 70% of cases, and frequently presents during childhood or adolescence [2]. The underlying pathogenesis of FD involves somatic activating mutations in the GNAS gene, which encodes the alpha subunit of the stimulatory G protein (Gsα) [3]. Fibro-osseous replacement of bone can lead to pathologic fracture, especially in weight-bearing bones or the upper extremities in athletes. This mutation disrupts normal signalling pathways, leading to aberrant differentiation and proliferation of osteoblastic and stromal cells. The resulting fibro-osseous lesions can cause pain, deformity, fractures, and functional impairment. Clinical presentation varies widely, with some cases being asymptomatic and incidentally discovered during radiological investigations. Others present with bone pain, pathological fractures, and skeletal deformities [4].

Radiographically, FD typically appears as a radiolucent lesion with a ground-glass appearance due to the mixture of fibrous tissue and woven bone [5]. Advanced imaging techniques, such as computed tomography (CT) and magnetic resonance imaging (MRI), aid in the accurate lesion characterization and assessment of soft tissue involvement. Histopathological examination remains the gold standard for diagnosis, revealing irregularly shaped trabeculae of woven bone within a fibrous stroma. The histological appearance helps differentiate FD from other bone lesions, such as giant cell tumours or aneurysmal bone cysts [6]. Given the rarity of the condition and its heterogeneous presentation, a multidisciplinary approach involving orthopaedic surgeons, radiologists, and pathologists is crucial for accurate diagnosis and optimal management.

The primary approach to treating FD involves surgery. The surgical approach, as described by Enneking and Gearen, entails the removal of the lesion through curettage, followed by the use of a bone graft to fill the void, aiming to prevent fractures and deformities [7]. However, anti-resorptive medications like bisphosphonates or anti-receptor activators of nuclear factor-kappa B ligand (anti-RANKL) antibodies are utilized to reduce the heightened bone turnover in FD management. This intervention has the potential to curtail or halt the enlargement of lesions, manage symptoms, and lower the risk of deformities and fractures, especially when diagnosed in its early stages. Dheenadhayalan et al. published an extensive manual on the treatment of FD with shepherd's crook deformity accompanied by a fractured femoral neck [8]. FD can also be associated with aneurysmal bone cyst [9,10]. This case report aims to contribute to the existing literature by presenting a detailed case of monostotic FD. We will highlight the clinical, radiographic, and histopathological aspects of the disease as well as discuss the management approach undertaken in this specific case.

## Case presentation

A 25-year-old male presented with a chief complaint of left hip pain over the past one and a half months. The patient was alright till one and a half months ago when he started experiencing insidious onset pain over the left hip which was gradually progressive. He reports no history of trauma or significant medical conditions. The patient describes the pain as a dull ache that is localized to his left hip and increased with movement of the limb and was relieved with rest and medication. Initially, the pain began as a mild discomfort but has progressively worsened over the last 15 days, limiting his ability to walk long distances and perform daily activities, and since the last two days, the patient has not been able to weight bear and walk on the affected lower limb. There is no radiation of pain to other areas. He denies experiencing any night sweats, weight loss, or fever. There is no history of joint swelling, joint stiffness, or neurological symptoms such as numbness or tingling. The patient's past medical history is unremarkable, with no known family history of bone disorders.

**Figure 1:**
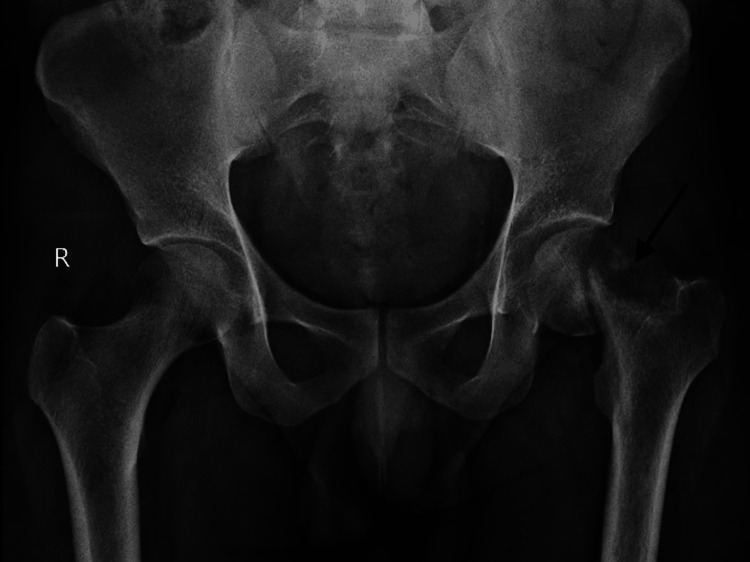
X-ray of the pelvis with both hips showing a sub-capital femoral neck fracture on the left side

The patient consulted multiple local practitioners for his above symptoms but his symptoms persisted and increased with time. Upon examination, the patient's gait could not be examined as he was not bearing weight on the left lower limb. The patient was examined in a supine position, and the left lower limb was externally rotated. Local inspection of the left hip revealed normal skin with no sinus, swelling, scars, or deformity. Palpation of the left femur elicited no local rise in temperature, and tenderness was present over the anterior joint line. The range of motion of the left hip could not be elicited due to pain. Distal neurovascular assessment of the left lower extremity revealed intact sensation, normal muscle strength, and symmetrical pulses. There were no signs of sensory deficits or motor weakness. Capillary refill time was within normal limits. X-ray of the pelvis with both hips showed a sub-capital femoral neck fracture on the left side (Figure 1). 

MRI of both hip joints revealed an irregular T2 hyperintense and T1 hypointense lesion involving the left femoral neck, with a few tiny cystic spaces seen within the lesion. Part of the lesion was extending into the superior-lateral aspect of the femoral head and surrounding bone marrow edema with minimal left hip joint effusion, features suggestive of a primary bony tumour. Plain CT of the hip joint and pelvis suggested an expansile lytic lesion with thin bony septation within and thick sclerotic margin in the left femoral head and greater trochanter, associated with sub-capital femoral neck fracture, suggestive of a giant cell tumour. The patient was managed with a bone biopsy from the femoral neck on the right side and was positioned supine on a fracture table after spinal anaesthesia. Under all aseptic conditions, cleaning, painting, and draping of the left lower limb were done. A 5 cm incision was made distal to the greater trochanter under C-arm guidance. The skin and soft tissue were dissected. Three guidewires were inserted towards the head of the femur in postero-superior, postero-anterior, and central directions. Multiple drilling was done with a drill bit. With the help of the drill bit, the bone was taken out and sent for biopsy to the histopathology lab. Thorough washing was given, closure was done in layers, sterile dressing was done, and the procedure was uneventful. The patient was then shifted to the post-op ICU. The core biopsy histopathology report came as fibrous tissue containing irregularly arranged bony trabeculae. Within the fibrous stroma, there's low cellularity, featuring unremarkable spindle-shaped fibroblasts set in a dense collagen matrix displaying a pattern resembling whorls or a storiform arrangement. The nuclei of the cells appear benign without significant atypia and mitosis. The cells surround trabeculae of woven bone which are short and irregular with curvilinearity. These trabeculae show no peripheral osteoblastic activity, suggestive of FD of the left femoral neck with no evidence of malignancy. The patient was then managed with a total hip replacement on the left side under spinal and epidural anaesthesia.

**Figure 2:**
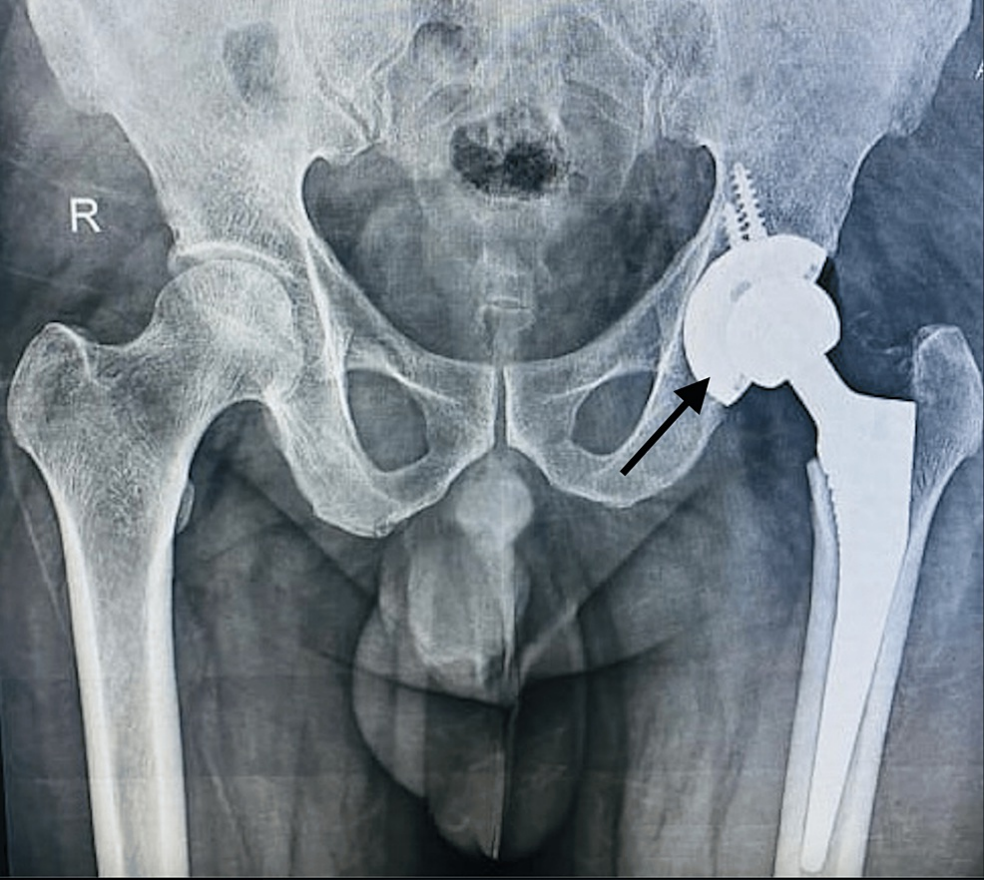
X-ray of the pelvis with both hips with total hip arthroplasty on the left side

Under all aseptic precautions, the patient was positioned in a right lateral position on the operating table. Under combined spinal and epidural anaesthesia, cleaning, painting, and draping were done. A 10 cm curved incision was made over the posterior edge of the greater trochanter. The skin, superficial fascia, and deep fascia were dissected. The fascia lata was incised, and the gluteus maximus was split along its fibers. The hip was rotated internally to expose the rotators and they were tagged. The capsule was identified, tagged, and incised in a T-shaped fashion. With traction and internal rotation, the hip was dislocated, and the fractured femoral head was removed. An eggshell-thin femoral head was noted. Gross fibrous tissue was noted around the femoral neck. The femoral head, bone tissue from the femoral neck, and fibrous tissue around the femoral neck were sent for histopathology. Soft tissue from the acetabular cavity was curetted. The acetabular cavity was prepared, and serial reaming was done followed by acetabular cup insertion. The femoral canal entry point was made, and the canal was prepared with a rasp. The femoral canal was prepared, and a trial of the femoral stem and head was taken and found to be satisfactory. Thorough washing was given. The femoral stem of size 02/135 was inserted to the femoral canal, and the femoral head size of 32mm was used. Washing was given, the head was reduced, and stability was confirmed and found to be satisfactory. The capsule was closed and rotators were sutured with Vicryl 1-0 (Aspiron, Meril Endo Surgery, Vapi, Gujarat, India). Washing was given with normal saline. A drain was inserted and fixed. The wound was closed with Vicryl 1-0 and Ethilon 2-0 (Aspiron, Meril Endo Surgery, Vapi, Gujarat, India). Sterile dressing was done. The procedure was uneventful, and the patient was shifted to ortho ICU. Sterile postoperative day 2 dressing was done. The suture site was healthy and dry. The post-op X-ray was satisfactory (Figure 2).

Postoperatively, the patient was started on physiotherapy in the form of static quadriceps and hamstring strengthening exercises, ankle pumps, knee range of motion exercises, and assisted full weight bearing. Suture removal was done on postoperative day 12. On discharge, the patient's vitals were stable, the surgical site was healthy and dry, full weight-bearing mobilization was initiated, active toe and ankle movements were encouraged, and distal circulation was intact.

## Discussion

Monostotic FD primarily affects long bones, ribs, and craniofacial bones. Radiographically, it presents as a ground-glass lesion with a lack of well-defined margins. FD is characterized by its chronic and often progressive nature. While lesions may reach a point of stabilization and cease further growth, they do not resolve entirely. The treatment of FD has changed over time, and it is now known that both basic curettage and curettage using an autogenous cancellous bone graft carry a significant recurrence risk. The graft of normal bone is gradually replaced by dysplastic bone as internal repair and remodelling take place, and the cavity frequently eventually returns to its preoperative state [11]. Most of the monostotic lesions stabilize with skeletal maturity [12]. Patients with FD necessitate a multidisciplinary approach involving physicians and surgeons for effective treatment [13]. In cases of the polyostotic form and growing children, individual lesions may exhibit a more accelerated rate of progression. Histologically, FD exhibits irregularly shaped trabeculae of woven bone interspersed within the fibrous stroma. Malignant transformation in FD is exceedingly uncommon, affecting less than 1% of cases. Differential diagnoses include other bone lesions such as giant cell tumours, aneurysmal bone cysts, and osteoblastoma. Hence, early diagnosis and management is of most importance. 

## Conclusions

Monostotic FD is a rare benign bone disorder that can present with various clinical manifestations. Timely diagnosis through a combination of clinical, radiographic, and histopathological assessments is crucial. Management should be tailored to the patient's symptoms and the potential for complications. Further research is warranted to explore the long-term outcomes of different treatment strategies and to enhance our understanding of the underlying pathophysiology of this condition.
